# Supplementary material for: Paralemmin-1 controls the nanoarchitecture of the neuronal submembrane cytoskeleton
Source: Sci Adv. 2025 Mar 7;11(10):eadt3724. doi: 10.1126/sciadv.adt3724 (PMC11887803; doi:10.1126/sciadv.adt3724)
Supplement: Supplementary file 3 — Data S1 and S2 [file sciadv.adt3724_data_s1_and_s2.zip › adt3724_data_s2.PDF]

Coding sequence of mEGFP-Palm1 generated by CRISPR/Cas9 knock-in

Exon 1 + 2bp exon 2 Palm1, gRNA Linker mEGFP Linker 3bp inverse gRNA, Palm1 exons 2-9

Nucleotide sequence

ATGGAGGCCCTGCATATAGGAGACTTCGCCATGGCTAGCGGAGTGAGCAAGGGCGAGGAGCT
GTTCACCGGGGTGGTGCCCATCCTGGTCGAGCTGGACGGCGACGTAAACGGCCACAAGTTCA
GCGTGTCCGGCGAGGGCGAGGGCGATGCCACCTACGGCAAGCTGACCCTGAAGTTCATCTGC
ACCACCGGCAAGCTGCCCGTGCCCTGGCCCACCCTCGTGACCACCCTGACCTACGGCGTGCA
GTGCTTCAGCCGCTACCCCGACCACATGAAGCAGCACGACTTCTTCAAGTCCGCCATGCCCGA
AGGCTACGTCCAGGAGCGCACCATCTTCTTCAAGGACGACGGCAACTACAAGACCCGCGCCGA
GGTGAAGTTCGAGGGCGACACCCTGGTGAACCGCATCGAGCTGAAGGGCATCGACTTCAAGGA
GGACGGCAACATCCTGGGGCACAAGCTGGAGTACAACTACAACAGCCACAACGTCTATATCATG
GCCGACAAGCAGAAGAACGGCATCAAGGTGAACTTCAAGATCCGCCACAACATCGAGGACGGC
AGCGTGCAGCTCGCCGACCACTACCAGCAGAACACCCCCATCGGCGACGGCCCCGTGCTGCT
GCCCGACAACCACTACCTGAGCACCCAGTCCAAGCTGAGCAAAGACCCCAACGAGAAGCGCGA
TCACATGGTCCTGCTGGAGTTCGTGACCGCCGCCGGGATCACTCTCGGCATGGACGAGCTGTA
CAAGGGCTCGAGCCCATCAACAAGTTTGTACAAAAAAGCAGGCTCCGCGGCCGCCCCCTTCAC
CGCCAGGATCCTGGCGACAGACACTGTGTCCCAGCAGGAGCGGCTTCAAGCAATCGCTGAGAA
GCGTAGGAAGCAGGCAGAGATTGAGAGCAAGCGGAGACAATTGGAGGATGACCGAAGACAGCT
GCAGTACCTGAAGTCCAAGGCACTTCGGGAACGCTGGCTGCTGGAGGGGACACCATCCTCAGC
CTCGGAGGGCGACGAGGACATGAGGAAGCAGATGCAGGAGGATGAACAGAAAGCCCGGAGCC
TGGAGGAGTCCATCACCAGACTCGAGAAAGAAATTGATGTCCTAGAGTTCGGTGAGTCAGCCCC
AGCTGCCCCAAAGGAGAACTCAGCGGCCCCCAGCCCCATCCGGCCCCACTCTACAAGCCCCG
CCAAGGAGGAGCAAAAGTCAGAAACCATGGTGAACGCCCAGCAGACTCCACTGGGCACCCCAA
AAGAGAATCGAAAGTCCACACCGGTGCGGAGTCCCGGGGGATCCACGATGATGAAGGCAGCCA
TGTATTCGGTGGAGATCACGGTGGAGAAGGACAAGGTGACCGGGGAGACCAGGGTGCTGTCC
AGCACCACATTGCTCCCCCGGGACCCACTCCCTCAGGGCGTGAAAGTCTACGAGGACGAAACA
AAAGTGGTCCATGCCGTGGACGGCCTCTCTGAGAACGGAATCCAGCCTCTAAGTTCCTCCGAG
GTGGATGAACTCATTCACAAGGCCGATGAGGTCACACTGAGTGAGGCCGGGTCCACAACTGGG
CCAGCAGAGCCTCGGGGACTCGCAGAGGATGTCACCAGGACCACACCATCCAGAAGGGAGAT
CACAGGAGTCGAGGCTCAGCCAGGAGAGGCCACGTCAGGCCCGCCAGGCATCCAGCCCGGTC
AGGAGCCCCCGGTCACCATGGTCTTCATGGGTTATCAGAATGTGGAAGATGAAGCAGAGACCAA
GAAGGTACTCGGCCTGCAGGACACCATCAAGGCTGAACTGGTGGTGATTGAAGACTCGGTCAC
CCCCAGGGAGCCTGCACCACTCAATGGCAGCGCGGCTGAGCTCCCAGCCACCAAGGAGGAGA
ACCAGACGGGGCCCACGACCACACCCAGCGACACCCAAGATCTTGACATGAAGAAGCCTCGCT
GTAGATGCTGTTCTGTCATGTGA

AA sequence

MEALHIGDFAMASGVSKGEELFTGVVPILVELDGDVNGHKFSVSGEGEGDATYGKLTLKFICTTGKL
PVPWPTLVTTLTYGVQCFSRYPDHMKQHDFFKSAMPEGYVQERTIFFKDDGNYKTRAEVKFEGDTL
VNRIELKGIDFKEDGNILGHKLEYNYNSHNVYIMADKQKNGIKVNFKIRHNIEDGSVQLADHYQQNTPI
GDGPVLLPDNHYLSTQSKLSKDPNEKRDHMVLLEFVTAAGITLGMDELYKGSSPSTSLYKKAGSAAA
PFTARILATDTVSQQERLQAIAEKRRKQAEIESKRRQLEDDRRQLQYLKSKALRERWLLEGTPSSAS
EGDEDMRKQMQEDEQKARSLEESITRLEKEIDVLEFGESAPAAPKENSAAPSPIRPHSTSPAKEEQK
SETMVNAQQTPLGTPKENRKSTPVRSPGGSTMMKAAMYSVEITVEKDKVTGETRVLSSTTLLPRDP
LPQGVKVYEDETKVVHAVDGLSENGIQPLSSSEVDELIHKADEVTLSEAGSTTGPAEPRGLAEDVTRT
TPSRREITGVEAQPGEATSGPPGIQPGQEPPVTMVFMGYQNVEDEAETKKVLGLQDTIKAELVVIED
SVTPR EPAPLNGSAAELPATKEENQTGPTTTPSDTQDLDMKKPRCRCCSVM
